# Unique Immune Gene Expression Patterns in Bronchoalveolar Lavage and Tumor Adjacent Non-Neoplastic Lung Tissue in Non-Small Cell Lung Cancer

**DOI:** 10.3389/fimmu.2018.00232

**Published:** 2018-02-12

**Authors:** Chih-Hsi Scott Kuo, Chien-Ying Liu, Stelios Pavlidis, Yu-Lun Lo, Yen-Wen Wang, Chih-Hung Chen, How-Wen Ko, Fu-Tsai Chung, Tin-Yu Lin, Tsai-Yu Wang, Kang-Yun Lee, Yi-Ke Guo, Tzu-Hao Wang, Cheng-Ta Yang

**Affiliations:** ^1^Division of Lung Cancer and Interventional Bronchoscopy, Department of Thoracic Medicine, Chang Gung Memorial Hospital, Taipei, Taiwan; ^2^Department of Computing, Imperial College London, Data Science Institute, London, United Kingdom; ^3^Division of Airway Diseases, Department of Thoracic Medicine, Chang Gung Memorial Hospital, Taipei, Taiwan; ^4^Division of Thoracic Medicine, Taipei Medical University Shuang Ho Hospital, New Taipei City, Taiwan; ^5^Genomic Medicine Research Core Laboratory, Chang Gung Memorial Hospital, Linkou, Taiwan

**Keywords:** lung cancer, non-small cell lung cancer, gene expression, differential expression, non-neoplastic lung tissue

## Abstract

**Background:**

The immune cells in the local environments surrounding non-small cell lung cancer (NSCLC) implicate the balance of pro- and antitumor immunity; however, their transcriptomic profiles remain poorly understood.

**Methods:**

A transcriptomic microarray study of bronchoalveolar lavage (BAL) cells harvested from tumor-bearing lung segments was performed in a discovery group. The findings were validated (1) in published microarray datasets, (2) in an independent group by RT-qPCR, and (3) in non-diseased and tumor adjacent non-neoplastic lung tissue by immunohistochemistry and in BAL cell lysates by immunoblotting.

**Result:**

The differential expression of 129 genes was identified in the discovery group. These genes revealed functional enrichment in Fc gamma receptor-dependent phagocytosis and circulating immunoglobulin complex among others. Microarray datasets analysis (*n* = 607) showed that gene expression of BAL cells of tumor-bearing lung segment was also the unique transcriptomic profile of tumor adjacent non-neoplastic lung of early stage NSCLC and a significantly gradient increase of immunoglobulin genes’ expression for non-diseased lungs, tumor adjacent non-neoplastic lungs, and tumors was identified (ANOVA, *p* < 2 × 10^−16^). A 53-gene signature was determined with significant correlation with inhibitory checkpoint *PDCD1* (*r* = 0.59, *p* = 0.0078) among others, where the nine top genes including *IGJ* and *IGKC* were RT-qPCR validated with high diagnostic performance (AUC: 0.920, 95% CI: 0.831–0.985, *p* = 2.98 × 10^−7^). Increased staining and expression of IGKC revealed by immunohistochemistry and immunoblotting in tumor adjacent non-neoplastic lung tissues (Wilcoxon signed-rank test, *p* < 0.001) and in BAL cell lysates (*p* < 0.01) of NSCLC, respectively, were noted.

**Conclusion:**

The BAL cells of tumor-bearing lung segments and tumor adjacent non-neoplastic lung tissues present a unique gene expression characterized by IGKC in relation to inhibitory checkpoints. Further study of humoral immune responses to NSCLC is warranted.

## Introduction

Novel immunotherapies focusing on immune checkpoint proteins have yielded significant progress in the treatment of advanced non-small cell lung cancer (NSCLC) in the past few years (1, 2). Treatment strategies of this sort mainly attempt the reconstitution of host immune surveillance that is otherwise suppressed by cancers, where the monoclonal antibodies targeting on programmed cell death 1 (PD-1) and PD-1 ligand (PD-L1) are by far the most successful instance.

In comparison to standard chemotherapy, a significant survival benefit was noted in previously treated advanced NSCLC patients receiving anti-PD-1 antibody (3, 4), suggesting that PD-1-mediated T cell exhaustion is a major factor in cancer progression. Relatedly, tumor expression of PD-L1 has recently been proposed as a predictive biomarker; however, given the manifold patterns of tumor microenvironments in terms of the abundance, constitution, and spatial distribution of the tumor-infiltrating immune cells, it is comprehensible that this marker only partially captures the dynamic picture of immune anergy during the development of NSCLCs, which is suggested by a number of studies showing that the prognostic value of PD-L1 in lung cancer is inconsistent (5–8).

In the meantime, ample evidences from both murine model and human tumor tissues have revealed that a dual pattern of inflammation around tumors can be recognized conceptually (9). This distinctive presentation in the two ends showing either abundant or sparse immune cells infiltration, appears to play a pivotal role not only in the prognosis of NSCLCs but also as a means of understanding the complex interactions between tumors and their neighboring environments (10). A wealth of knowledge has shown that lung-resident macrophages, which are frequently found to constitute a major component of tumor-infiltrating immune cells, poise to tip the balance toward either classical or non-classical activation depending on their engagement with dominant signals in tumor milieu (11, 12). Three types of non-classically activated macrophages that exhibit immunoregulatory properties characterized by different transcription factors, gene expression repertoires, and effector cytokines are currently recognized (13), and their respective prognostic values in the context of NSCLS have been established (14).

Tumor-infiltrating lymphocytes, on the other hand, also exert significant impacts on tumor development (15). Some subpopulations of these lymphocytes have been shown to be prognostic for a number of solid cancers, with their prognostic values presumptively due in some degree to their relative abundances in tumor milieus. In this regard, CD4^+^Foxp3^+^ T cells have been shown to suppress antitumor immunity (16), whereas CD8^+^ T cells, CD8^+^CD45RO^+^ cells, and CD8^+^CD103^+^ memory T cells have been found to play roles in antitumor activities (17–19). Recently, emerging evidence has indicated that a similar pro- and antitumor immune framework also describes the actions of B lymphocytes (20). The regulatory properties exhibited by various subsets of CD5^+^ and CD24^+^ B cells may be attributed to their IL-10 or TGF-β-producing capacities and also to their surface PD-L1 expressions (21–23). In contrast, CD20^+^ B cells may, in tumor microenvironments, act as antigen-presenting cells that work in collaboration with CD8^+^ T cells to form tertiary lymphoid structures, which are associated with a favorable prognosis in solid tumors (24, 25). In the context of NSCLC, CD138^+^ tumor-infiltrating plasma cells that express immunoglobulin kappa C, rather than CD20^+^ B cells, have been reported as a favorable prognostic marker (26).

Meanwhile, accumulated evidences have suggested that these tumor-infiltrating immune cells have key implications to allow successful immunotherapeutic approaches (27); however, their isolation from human advanced NSCLC poses a major hurdle. In this regard, bronchoalveolar lavage (BAL) cells from the alveolar milieus of tumor-bearing lung segments may serve as an alternative for the investigation of immunological characteristics in response to tumors. In this study, we sought to explore the transcriptomic profiles of BAL cells taken from a discovery group consisting of both advanced NSCLC patients and healthy controls using transcriptomic microarray with the aim of defining their characteristic gene expression pattern. Validation of the findings was carried out using published microarray datasets and through verification in a second independent group of study subjects.

## Materials and Methods

### Study Subjects and Design

**Figure 1:**
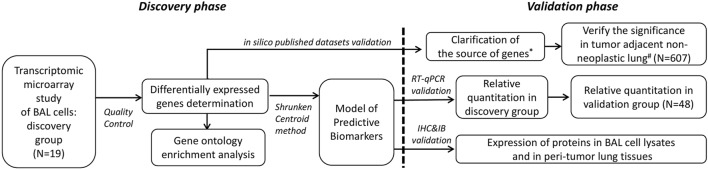
Demonstration of the two-phase workflow of the study. *Datasets of Gene Expression Omnibus (GEO) accession: GSE10245, GSE32474, GSE1133, GSE72642, and GSE8823 were used. ^#^Datasets of GEO accession: GSE1643, GSE16538, GSE24206, GSE18995, GSE19188, GSE19804, GSE40791, and GSE27262 were used. RT-qPCR, reverse transcription quantitative polymerase chain reaction; IHC, immunohistochemistry; IB, immunoblotting.

Two groups of participants were enrolled separately in this study. This included the first group of 13 advanced NSCLC subjects and 6 healthy controls for discovery purpose using transcriptomic microarray analysis. The major findings attained in the discovery group were attempted for published microarray datasets validation and for biological verification in the second independent group of 34 advanced NSCLC subjects and 14 healthy controls along with the analysis of protein expression by immunohistochemistry in resected lung specimens and immunoblotting in cell lysates of BAL (Figure 1). The study was approved by the Ethics Committees of Chang Gung Memorial Hospital, Linkou, and all the participants gave written and signed informed consent.

### Bronchoalveolar Lavage

Bronchoalveolar lavage specimens were collected during routine diagnostic bronchoscopy and BAL was performed from the lung segment in which tumor was located and from right middle lobe or lingular lobe in the normal controls (NC) *via* instillation of 250 c.c. of saline fluid to harvest the cells. The BAL samples were immediately preserved at 4°C, and the cells were pelleted within 1.5 h *via* 300 × *g* centrifugation for 15 min. An aliquot of cells from each sample was separated for the determination of differential cell counts using a Cytospin™ 4 Cytocentrifuge (Thermo Fisher Scientific, Taiwan). The rest of the cells were immediately lysed in buffer RLT and preserved at −80°C for the later RNA extraction.

### RNA Extraction and Microarray Analysis

Total RNA was isolated from each of the BAL samples using RNeasy mini kits (QIAGEN, Valencia, USA), and the RNA purity and integrity were measured using a Nanodrop 1000 (Thermo Fisher Scientific, Taiwan) and Agilent Bioanalyser (Agilent, Santa Clara, CA, USA), respectively. All the RNA samples met the quality criteria of OD_260_/OD_280_ >2.0 and RNA integrity number >9.0. The extracted RNA was labeled with streptavidin-phycoerythrin conjugate and hybridized to an Affymetrix HG-U133 Plus 2.0 microarray (Affymetrix, Santa Clara, CA, USA) as recommended by the manufacturers. The raw data were quality assessed and preprocessed by robust multi-array average normalization using the Bioconductor R package affy. The gene expression levels were further adjusted by removing the unwanted effects from technical batches, sex, age, and smoking using the Bioconductor R package Surrogate Variable Analysis (*sva*).

### Quantitative Real-time Polymerase Chain Reaction

Total RNA (1 µg) was utilized for reverse transcription using the RevertAid First Strand cDNA Synthesis Kit (Thermo Fisher Scientific, Taiwan). Quantitative real-time PCR was performed using the Rotor-Gene 6000 Real-Time PCR System (Corbett-Research, Mortlake, Australia). Each 20 µl reaction contained 5 ng of cDNA, 10 µl of SsoFast EvaGreen Supermix (Bio-Rad, Taiwan), and a final concentration of 300 nM of forward and reverse primer. A set of 14 reported endogenous reference genes were examined for their expression levels in microarray data (Figure S1 in Supplementary Material). Following this approach, *HPRT1* was determined as the endogenous reference due to its highly constitutive expression in the samples from both the cancer and control subjects. The primer pairs of target genes included *IGJ, IGKC, CEACAM6, MMP7, SPP1, CXCL13, SLC40A1, CPA3*, and *YES1*, which were designed and examined for specificity using Primer-BLAST (Table S1 in Supplementary Material). The amplification efficiency of each primer pair was determined using the standard curve method by serial template dilution to six orders of magnitude. All the reactions were performed in duplicates and the double-delta cycle threshold (ΔΔCt) method was applied for relative quantitation in line with the minimum information for publication of quantitative real-time PCR experiments (MIQE) guidelines (28).

### Immunohistochemistry

Samples of formalin-fixed paraformaldehyde-embedded sections (4 µm) were dewaxed and rehydrated prior to heat-induced epitope retrieval using Tris-EDTA Buffer at pH 9.0. Primary antibodies of polyclonal rabbit anti-human kappa light chains/IGKC antibody (A0191, DAKO), polyclonal rabbit anti-human Immunoglobulin J chains/IGJ antibody (Atlas Antibodies, HPA044132, SIGMA-ALDRICH), and polyclonal rabbit anti-human CPA3 antibody (Atlas Antibodies, HPA008689, SIGMA-ALDRICH) were used. Visualization was performed using the Envision Mouse/Rabbit-HRP (DAKO) system and chromogen detection using 3-amino-9-ethylcarbazole (AEC).

### Western Blot Analysis

Aliquots of total protein (30 µg) were loaded with Laemmli sample buffer for electrophoresis using polyacrylamide gel with a 4–20% gradient, and polyvinylidene difluoride membranes were used for protein transfer. The membranes were blocked with protein-free SuperBlock blocking buffer (GeneStar, Taiwan) before overnight incubation with polyclonal rabbit anti-human kappa light chains/IGKC antibody at a dilution factor recommended by the manufacturers. Blots were developed using peroxidase substrate with enhanced chemiluminescence (Pierce™ ECL, Thermo Scientific) and later exposed to a UVP BioSpectrum 810 Imaging System™ (Cambridge, UK).

### Statistical Analysis and Predictive Model Construction

All categorical variables were analyzed using Fisher’s exact test, while Student’s *t*-test was used for comparisons of means between the two groups. A linear model (Bioconductor R package limma) with adjustments for technical batch, age, sex, and smoking was used for differentially expressed genes (DEGs) analysis, and an original *p*-value <0.05 was considered statistically significant in the discovery phase of the study. A predictive model was generated by the nearest shrunken centroid learning algorithm using the R package pamr as previously described (29, 30). This approach coupled with the 10-fold cross validation method generated a reduced set of genes/biomarkers determined by their accuracy in classifying the phenotype of interest.

## Results

### Clinical and Pathologic Characteristics

A total 47 advanced NSCLC patients and 20 NC were enrolled, and these study participants were assigned to either the discovery (*n* = 19) group or the validation (*n* = 48) group, with the two groups being balanced in terms of clinical and pathologic characteristics (Table 1). The BAL specimens subjected to cytospin preparation were routinely examined using standard morphology criteria, where similar cell distributions of macrophages (85%), lymphocytes (10–15%), and granulocytes (<5%) were identified for all the study subjects.

**Table 1:** Clinical and pathological characteristics of all study subjects.

	Discovery group			Validation group		
Variables	Non-small cell lung cancer (NSCLC) (*n* = 13)	Control (*n* = 6)	*p*-Value	NSCLC (*n* = 34)	Control (*n* = 14)	*p*-Value
**Age**	57.5 ± 11.3	52.3 ± 11.7	0.370	58.5 ± 12.8	53.3 ± 11.4	0.162
**Sex (female)**	5 (38.5)	4 (66.7)	0.516	15 (44.1)	7 (50.0)	0.958
**Smoking**	3 (23.1)	1 (16.6)	1.000	9 (26.5)	3 (21.4)	1.000
**Histology**						
Adenocarcinoma	9 (69.2)			26 (76.5)		0.892^b^
Squamous	4 (30.8)			8 (23.5)		
**EGFR status^a^**						
Wild type	3 (33.3)			11 (42.3)		0.520^b^
L858R	3 (33.3)			4 (15.4)		
19 deletion	3 (33.3)			8 (30.8)		
Others	0			3 (11.5)		
**Staging**						
IIIa	1 (7.7)			6 (17.6)		0.602^b^
IIIb	3 (23.1)			5 (14.7)		
IV	9 (69.2)			23 (67.7)		

*^a^Exclusively adenocarcinoma*.

*^b^Comparison of lung cancer patients between discovery and validation group*.

### Transcriptomic Profile of BAL Cells

**Figure 2:**
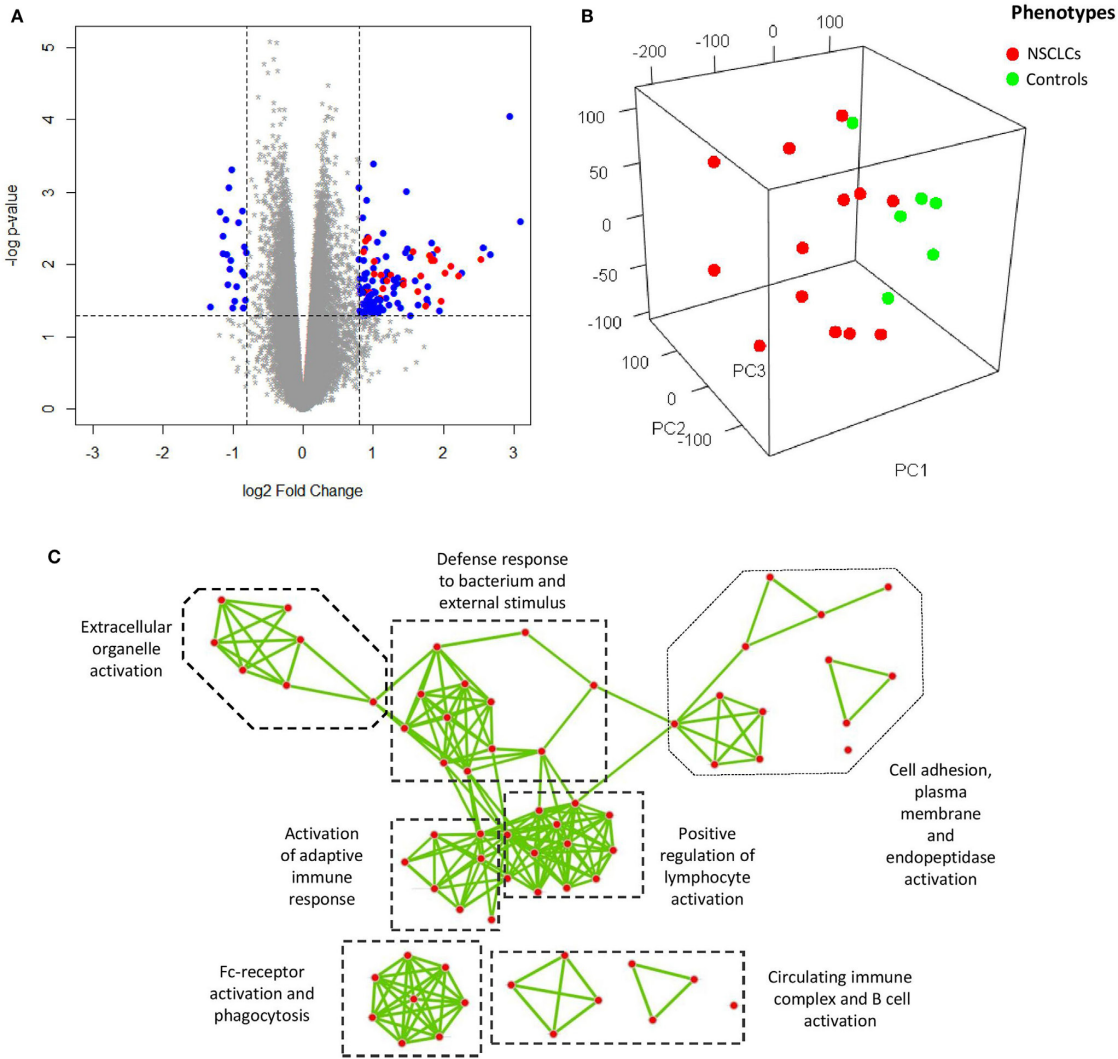
**(A)** Volcano plot showing differentially expressed genes (DEGs) (blue and red) of bronchoalveolar lavage cells. Overexpression of a subset of genes encoding immunoglobulin (red) is shown. **(B)** Visualization of the expression matrix by principle component analysis from all microarray probe sets of discovery group, where the advanced non-small cell lung cancer (NSCLC) patients (red) versus the controls (green) delineated the major source of variation along the principle component 1 (PC1). **(C)** Network visualization of gene ontology enrichment analysis for the DEGs. Each node represents an enriched GO-term and each edge represents the presence of overlapping genes between two GO-terms.

Principle component analysis (PCA) was used to visualize the processed microarray data, in which removal of the effects from technical batches was confirmed (Figure S2 in Supplementary Material), leaving cancer as the major source of variation for the whole expression matrix (Figure 2B). In our attempt to explore the characteristic transcriptomic profile of BAL cells from tumor-bearing lung segments, we first analyzed the DEGs of BAL cells between the advanced NSCLC patients and the NC in the discovery group, and a total 129 DEGs (log_2_ fold change >0.8 and nominal *p* < 0.05) were identified (Figure 2A). As a next step, we endeavored to understand their biological relevance. To this end, gene ontology enrichment analysis (GOEA) was performed on three publicly available ontology databases (GO, Reactome, and KEGG) in order to identify the links between the DEGs and specific functional categories. We combined network visualization for the enriched GO-terms using Cytoscape Enrichment Map plug-in (31) and found that the topology of gene enrichment was concentrated in a handful of functional domains, including Fc-receptor activation; the positive regulation of lymphocyte activation; humoral immunity and B cell activation; and extracellular matrix, cell adhesion, and proteinase activity (Figure 2C). These functional domains were represented by multiple pathways involving Fc gamma receptor-dependent phagocytosis, circulating immunoglobulin complex, endopeptidase activity, and extracellular matrix interactions (Table 2).

**Table 2:** Top 10 representative pathway enrichments of differentially expressed genes from public ontology databases.

Database ID	Name	*p*-Value
REAC:2029481	Fc gamma receptor (FCGR) activation	5.41 × 10^−11^
GO:0042571	Immunoglobulin complex, circulating	2.17 × 10^−10^
REAC:2029480	FCGR-dependent phagocytosis	6.70 × 10^−8^
REAC:2173782	Binding and uptake of ligands by scavenger receptors	1.35 × 10^−7^
GO:0006959	Humoral immune response	1.80 × 10^−6^
GO:0042113	B cell activation	2.00 × 10^−4^
GO:0046649	Lymphocyte activation	2.54 × 10^−3^
GO:0004175	Endopeptidase activity	3.02 × 10^−3^
GO:0008146	Sulfotransferase activity	1.50 × 10^−2^
KEGG:04512	ECM–receptor interaction	1.80 × 10^−2^

### Assessment of the Cell Lineage Specificity

**Figure 3:**
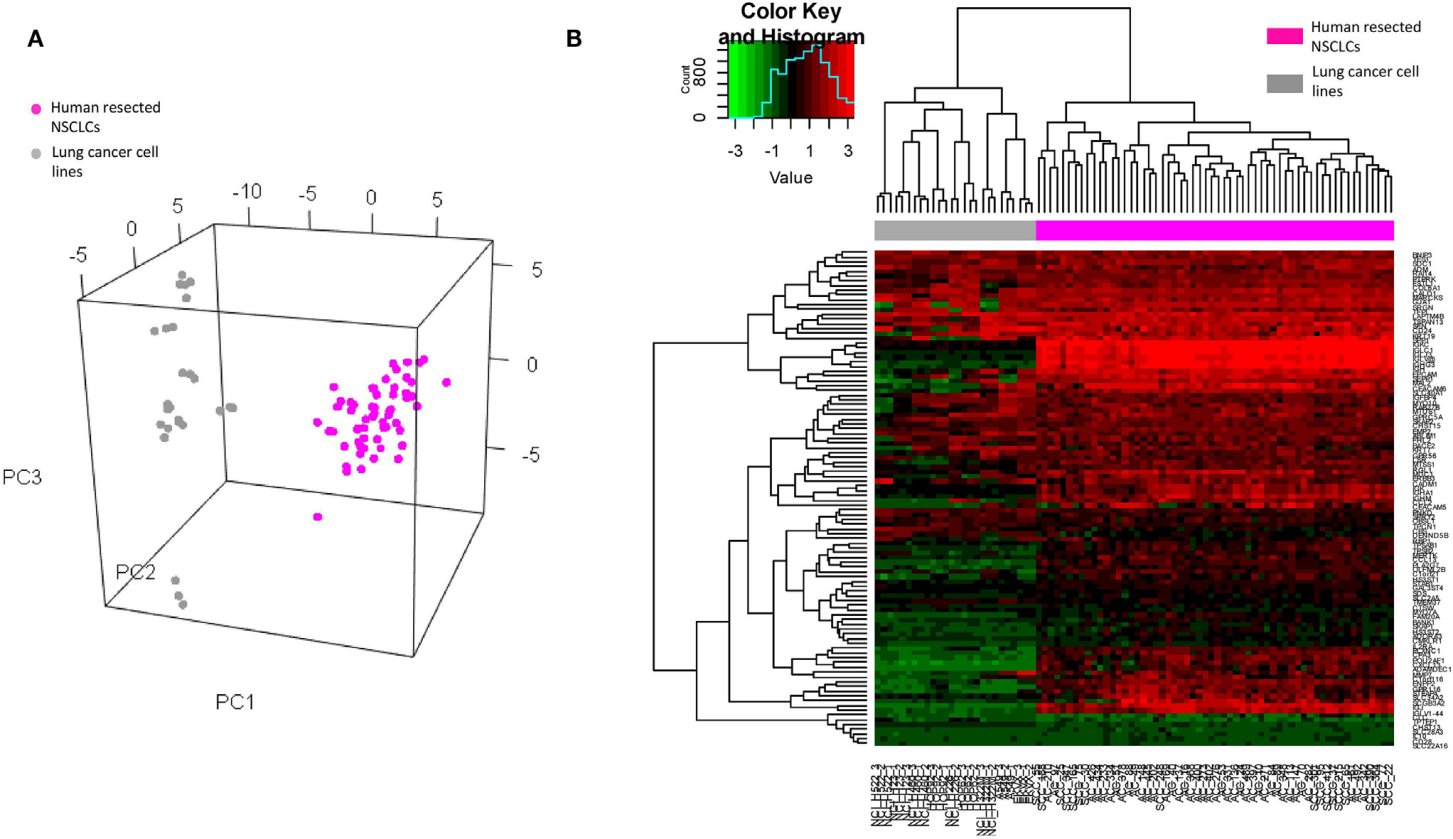
**(A)** Visualization of principle component analysis using the standardized expression matrix of differentially expressed genes of bronchoalveolar lavage cells extracted from the datasets GSE10245 (magenta, *n* = 58) and GSE32474 (gray, *n* = 26), which corresponded to the human resected non-small cell lung cancer (NSCLCs) and the nine lung cancer cell lines of the NCI-60 human tumor cell lines panel, respectively. **(B)** Unbiased clustering of the same expression matrix clearly differentiating resected tumors from cell lines.

To ascertain whether the DEGs could be credited to specific cell lineages, we subsequently carried out a number of *in silico* analyses on the published microarray datasets from the Gene Expression Omnibus. The original expression value of each dataset was transformed by standardization and mean-centered to enable comparative analysis (32). Given that the identified DEGs were from BAL cells of tumor-bearing lung segments, it was essential to clarify that the signals were not dominated by potentially contaminated cancer cells. To address this, these DEGs were extracted from microarray data of human resected lung cancer (33) tissue (which consisted of a mixture of malignant, matrix, and infiltrating immune cells, *n* = 58) and of 9 lung cancer cell lines of the NCI-60 human tumor cell lines panel (34) (which had high purity of malignant cells, *n* = 26) for PCA and clustering analysis. The results showed that the identified DEGs clearly separate the two datasets along the first principle component (Figure 3A) and also unambiguously assign them to different clusters (Figure 3B), suggesting that the source of the DEGs was most likely non-malignant cells. This finding was reinforced as a set of 14 genes from The Cancer Genome Atlas that specifically characterize malignant cells (35) prevented the discrimination between the resected tumors and the cell lines (Figure S3 in Supplementary Material).

Later, the DEGs were taken to interrogate the normal human tissue gene expression database from Su et al. (36). A tissue is defined as overexpressing a gene when its expression level of the gene is twice the SD above the mean for all tissue types. We found that the identified DEGs were significantly expressed in hematopoietic cell lineages, particularly CD19 B cells, CD33 myeloid cells, BDCA4 dendritic cells, and CD14 monocytes (Figure S4 in Supplementary Material). Hierarchical clustering on the other two datasets of fractionated blood (37) and BAL cells (38) of healthy controls also suggested alveolar macrophages, B lymphocytes, and granulocytes as the major sources of these DEGs (Figure S5 in Supplementary Material).

### Transcriptomic Profile of BAL Cells Is Unique during NSCLC Development

**Figure 4:**
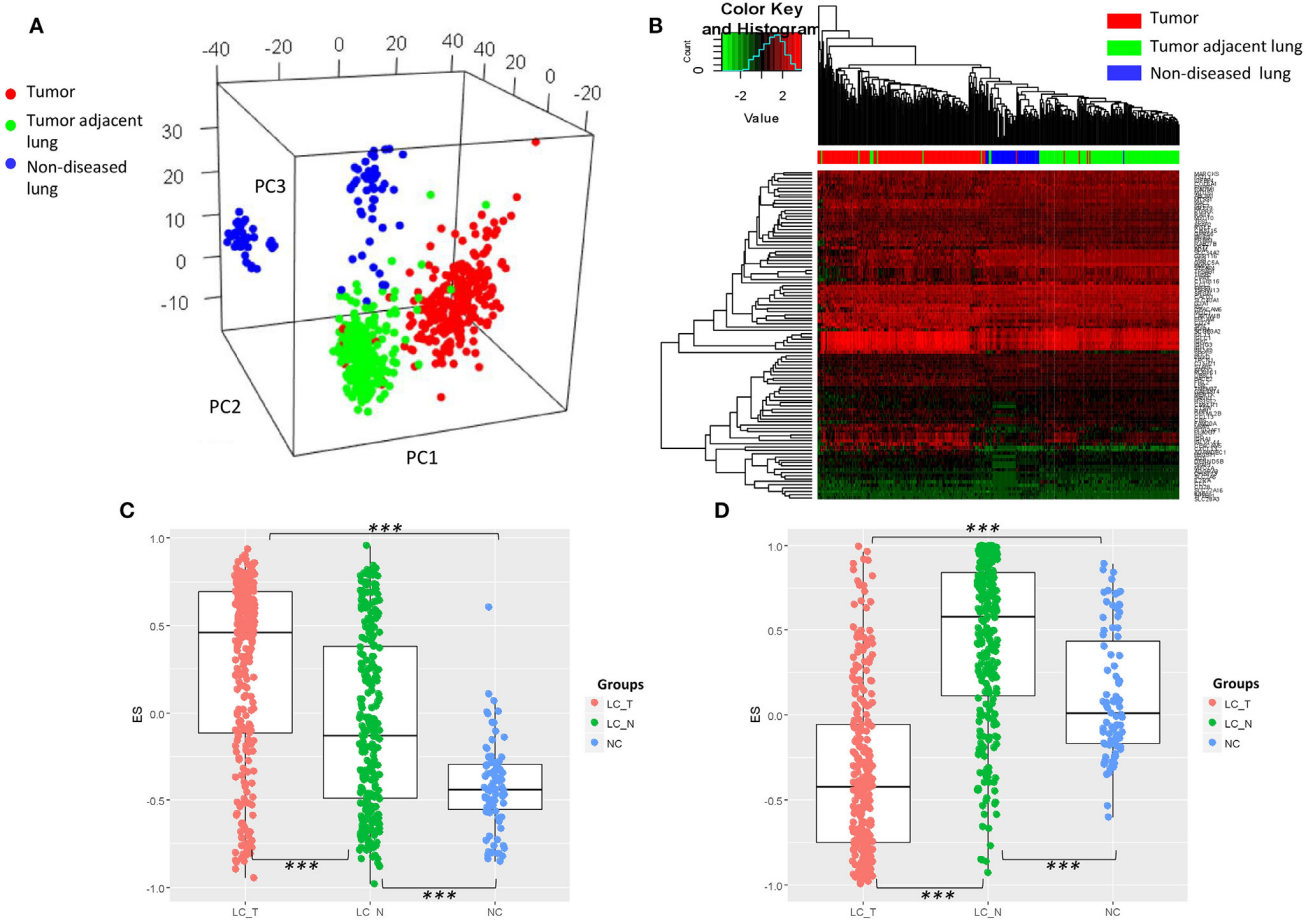
**(A)** Visualization of principle component analysis using the standardized expression matrix of differentially expressed genes of bronchoalveolar lavage cells extracted from eight datasets, which corresponded to the tissues of non-diseased lungs (*n* = 87, blue), the tissues of human resected tumors (*n* = 273, red), and the tissues of tumor adjacent normal lungs (*n* = 247, green). **(B)** Unbiased clustering using the same expression matrix showing clear distinctions between the three tissue types, except for one sample of non-diseased lung tissue. Expression of the gene set from **(C)** the composite of immunoglobulin genes and **(D)** the genes of mast cell proteinases *CPA3, TPSAB1*, and *TPSB2*. LC_T, resected tumor; LC_N, tumor adjacent normal lung; NC, normal control non-diseased lung, ***p < 2.0 × 10^−7^ by Tukey HSD test.

As the characteristic transcriptomic profile of BAL cells was noted in the advanced NSCLC patients, we endeavored to understand whether that profile could constitute a common feature that has happened earlier at the early stage of lung cancers. To this end, the expression values of the identified DEGs were extracted from eight published microarray datasets of early stage resected tumors (*n* = 273) and tumor adjacent normal lung tissues (*n* = 247) (39–42), as well as normal non-diseased lung tissues (*n* = 87) (43–46). The original expression value of each dataset was preprocessed by standardization and mean-centered to enable comparative analysis (32). PCA of the expression matrix showed three separated groups that clearly matched the various tissue types (Figure 4A) and unbiased clustering that also differentiated these tissue types from each other (Figure 4B), suggesting what appears histologically normal tumor adjacent lungs are otherwise immunologically different from non-diseased lungs and its paired tumor tissues. As a further step, using Gene Set Variation Analysis (GSVA) as previously described (29), we found that the gene set from the composite of immunoglobulin genes (Figure 1A) showed a gradient increase of expression all the way from the tissues of non-diseased lungs, to tumor adjacent lungs, to the tumors themselves (Tukey HSD test: *p* < 2.0 × 10^−7^ for each pair of comparisons, Figure 4C). In addition, a significantly high expression of the gene set of mast cell proteinase *CPA3, TPSAB1*, and *TPSB2* was also noted in the tumor adjacent lungs (Tukey HSD test: *p* < 2.0 × 10^−7^ for each pair of comparisons, Figure 4D).

### Signature Characteristic of BAL Cells

**Figure 5:**
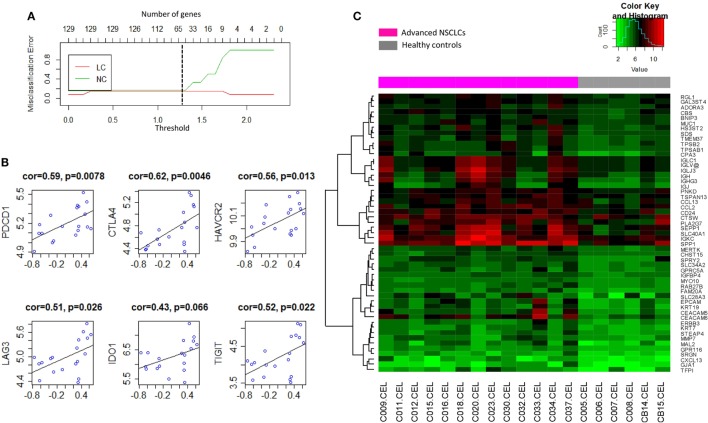
**(A)** Shrunken centroid method coupled with 10-fold cross-validation for determining the optimal number of genes for the signature model. A shrunken threshold of 1.25 corresponding to the reduction of 62 genes (black broken line) preserved the lowest error rate. **(B)** Correlation analysis of the signature of bronchoalveolar lavage (BAL) cells (*X*-axis) with the gene expression levels of checkpoint proteins programmed cell death 1/*PDCD1, CTLA4*, TIM-3/*HAVCR2, LAG3, IDO1*, and *TIGIT*. **(C)** Heat map visualization of the global expression for the 53 upregulated genes of BAL cells in the discovery group.

To determine the signature that best characterized the transcriptomic profile, the shrunken centroid algorithm coupled with 10-fold cross validation was applied as previously described (29, 30) in the discovery group. A model of the signature reduced to 62 genes (Table S2 in Supplementary Material) was identified with the classification accuracy at 83% (Figure 5A). Overall, 53 (85.5%) of those genes were found to be upregulated in NSCLC, and their global expression across all the subjects in the discovery group was displayed (Figure 5C). Interestingly, these 53 upregulated genes, summarized by the GSVA as a signature of BAL cells from tumor-bearing lung segments, showed moderately high correlations with the gene expressions of multiple checkpoint proteins (Figure 5B), including PD-1/*PDCD1* (*r* = 0.59, *p* = 0.0078), *CTLA4* (*r* = 0.62, *p* = 0.0046), TIM-3/*HAVCR2* (*r* = 0.56, *p* = 0.0130), *LAG3* (*r* = 0.51, *p* = 0.0260), *IDO1* (*r* = 0.43, *p* = 0.0660), and *TIGIT* (*r* = 0.52, *p* = 0.0220), where similar correlations, except for that with *CTLA-4*, were also noted in the published microarray datasets (Figure S6A in Supplementary Material). Furthermore, PCA of this signature presented a dichotomous gene expression pattern of these BAL cells from NSCLC (Figure S6B in Supplementary Material), indicating the existence of inter-patient heterogeneity.

### Validation of the Biomarker Findings

**Figure 6:**
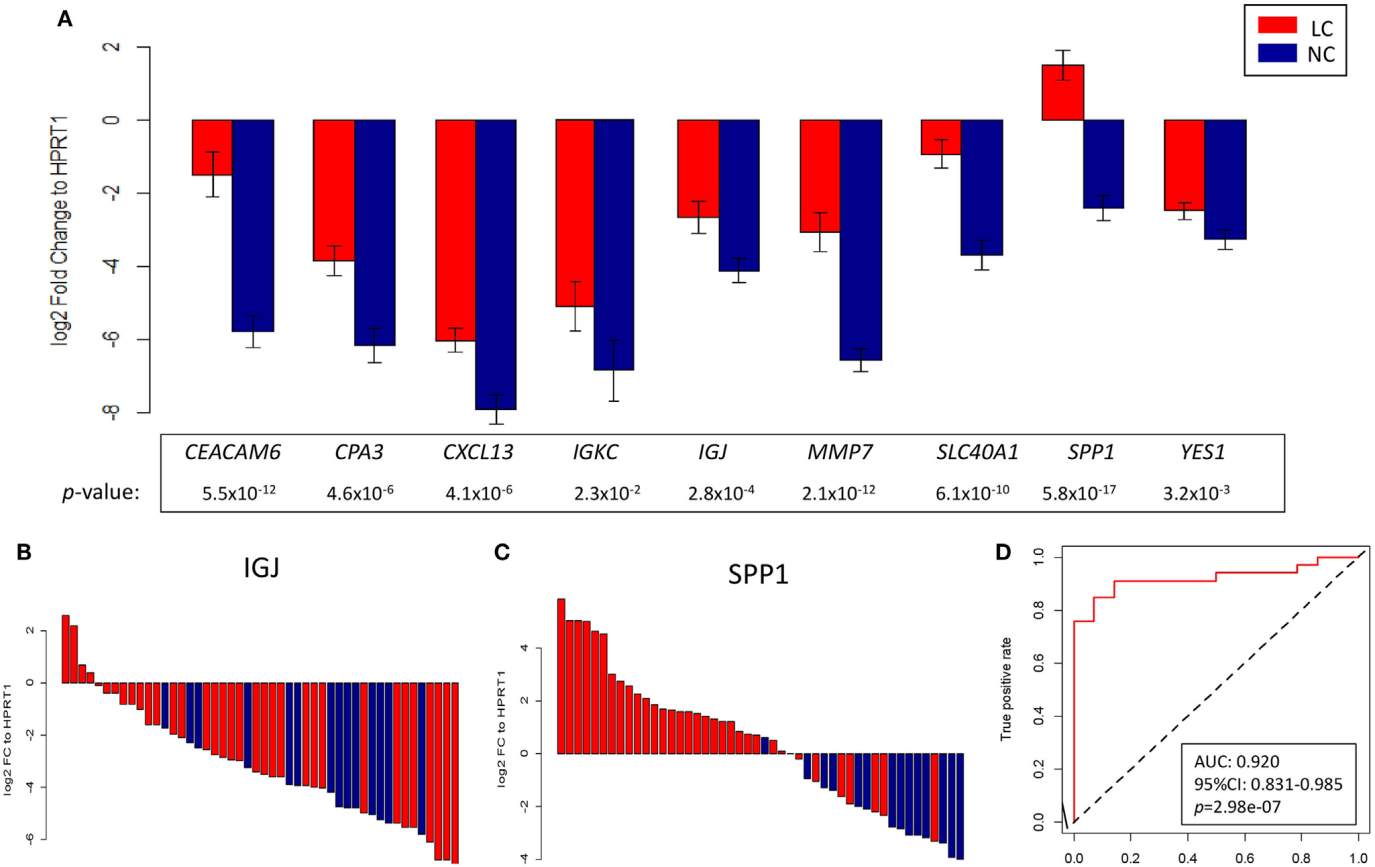
**(A)** Log2 fold change against *HPRT1* of each of the nine genes measured by RT-qPCR in the validation group between advanced non-small cell lung cancer (red) and control (blue) subjects. Representative waterfall plots of gene *IGJ* **(B)** and gene *SPP1* **(C)** showing the log2 fold change against *HPRT1* of each individual in the validation group. **(D)** ROC curve showing the differentiation performance of the nine genes in the validation group.

As a next step, validation of a handful of top-ranked and intriguing biomarkers was attempted. This included *SPP1*, which plays a role in cell–matrix interactions and cytokine activity; *CEACAM6*, a glycoprotein involved in cell adhesion and migration; *MMP7*, which encodes a member of the metalloproteinases associated with matrix breakdown; *SLC40A1*, which encodes a membrane protein functioning in iron metabolism; *IGJ*, which is responsible for an immunoglobulin J chain polypeptide; *IGKC*, which is responsible for a constant region in immunoglobulin kappa light chains; *CPA3*, which encodes carboxypeptidase A3 frequently released by mast cells; *YES1*, which is responsible for a Src family protein tyrosine kinase; and *CXCL13*, which encodes a B lymphocyte chemoattractant. Using *HPRT1* as an endogenous reference, RT-qPCR confirmed the overexpression of these genes in a manner consistent with the pattern found in the microarray study of the discovery group (Figure S7 in Supplementary Material). As an extension of this validation, we subsequently assessed the reproducibility of the expression pattern in an independent group of 34 NSCLCs and 14 NC, in whom we confirmed that the pattern of gene expression found in the discovery group was recapitulated (Figures 6A–C). These nine genes were later utilized in predictive model training using support vector machine in the discovery group, and the high performance of the resulting model was ascertained by ROC curve analysis (AUC: 0.920, 95% CI: 0.831–0.985, *p* = 2.98e–07; Figure 6D) when applied in the validation group.

### Protein Expression of Peri-Tumor Lung Tissue and BAL Cells

**Figure 7:**
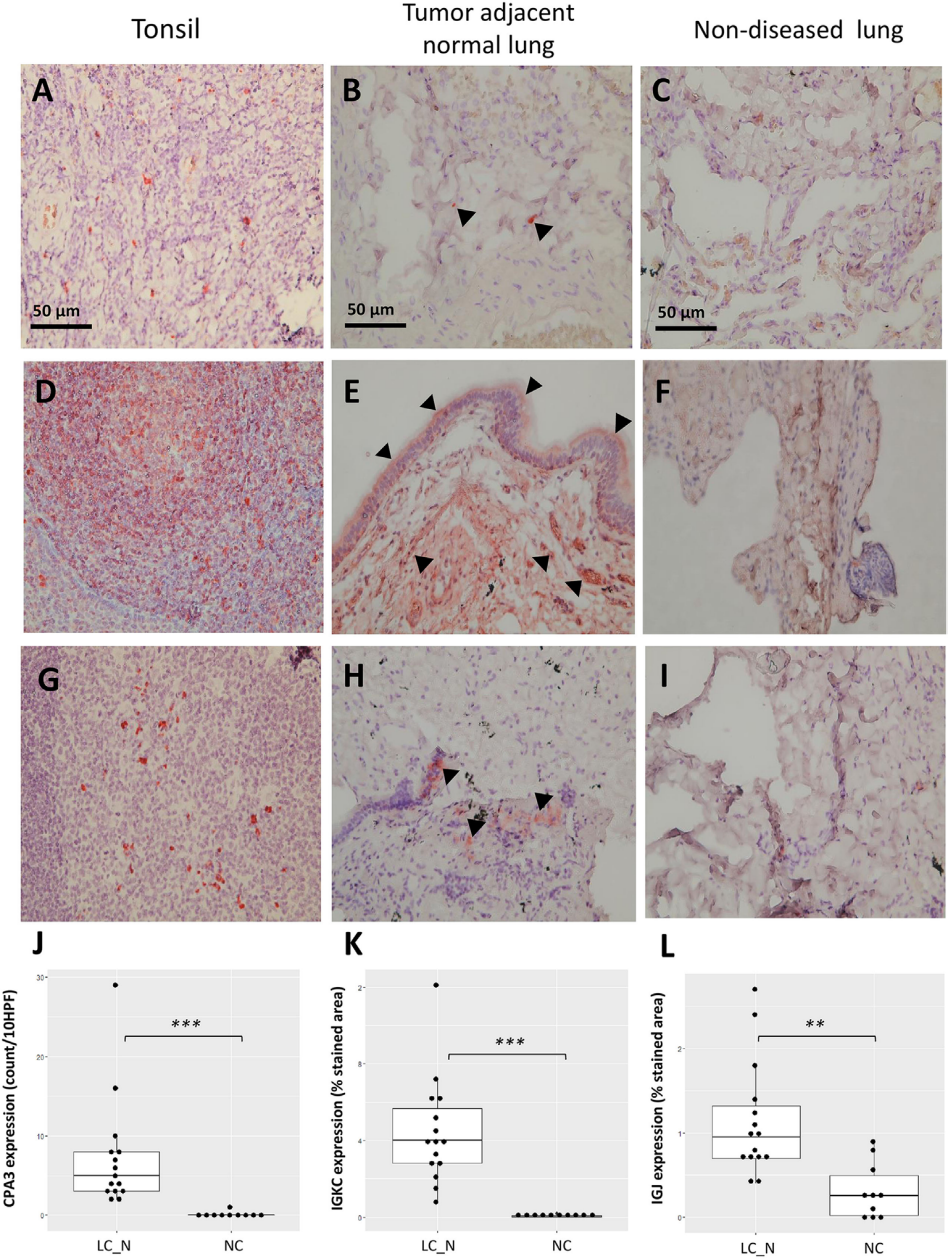
Representative immunohistochemistry staining of CPA3 **(A–C)**, IGKC **(D–F)**, and IGJ **(G–I)** from the positive control tonsil tissue, tumor adjacent normal lung tissue, and non-diseased lung tissue under 400× high power magnification, respectively. Increased staining of each protein was noted in the matrix of tumor adjacent normal lung tissue [arrowhead **(B,E,H)**]. Quantitative analysis of the expression of CPA3 **(J)**, IGKC **(K)**, and IGJ **(L)** between tumor adjacent normal lung tissue (LC_N, *n* = 15) and non-diseased lung tissue (normal controls, *n* = 10). ***p < 0.001 and **p < 0.01 by Wilcoxon signed-ranked test, HPF, high power field.

**Figure 8:**
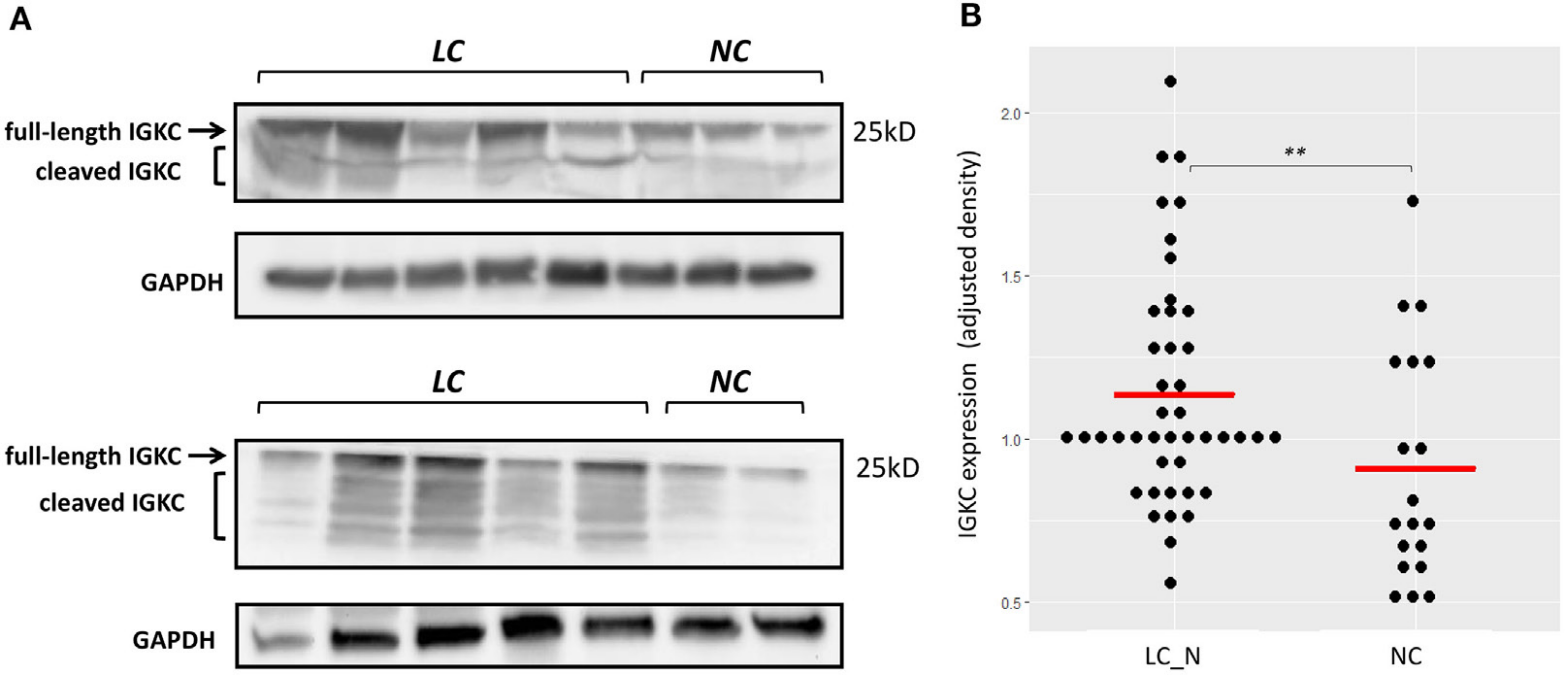
**(A)** Representative immunoblotting showing increased IGKC expression of the bronchoalveolar lavage (BAL) cell lysates of non-small cell lung cancer subjects (LC) compared to the BAL cell lysates of normal controls (NC). **(B)** Quantitative analysis of the expression of IGKC, density adjusted to GAPDH, between LC (*n* = 43) and NC (*n* = 19). ** *p* < 0.01 by Wilcoxon signed-ranked test.

Immunohistochemistry was later carried out for the validation of protein expression in early stage resected NSCLCs focusing on the immunoglobulins and mast cell carboxypeptidase A3, where tissues of tumor adjacent normal lungs and non-diseased lungs from surgical samples of pneumothorax patients being used. Significantly higher staining levels of the proteins IGKC (Wilcoxon signed-rank test, Figures 7D–F,K; *p* < 0.001), IGJ (Figures 7G–I,L; *p* < 0.01), and CPA3 (Figures 7A–C,J; *p* < 0.001) were noted in the tumor adjacent normal lung tissues. The increased staining levels of these proteins were more frequently found in the matrix tissue than in the alveolar air space of the lungs, particularly in the case of IGKC protein (Figure 7E). Immunoblotting also revealed that the lysates of BAL cells of NSCLC presented a significantly higher protein level of IGKC (Wilcoxon signed-rank test, *p* < 0.01), for both the full-length (25 kD) and the cleaved forms, than the lysates of BAL cells of NC (Figure 8).

## Discussion

This study showed that BAL cells of tumor-bearing lung segments displayed a characteristic transcriptomic pattern as a result of the milieu effect of the tumors. In fact, tumor adjacent normal tissues of multiple types of solid tumors have recently been reported not only to constitute an intermediate state between non-tumor bearing normal and tumor tissues but also a distinct tissue phenotype in terms of the transcriptomic alterations linked to pro-inflammatory pathways, multiple immune cells, and endothelial cells (47). Furthermore, *in silico* analysis revealed that the transcriptomic profile of BAL cells from advanced NSCLC was also a hallmark of tumor adjacent non-neoplastic lung tissues in early stage lung cancers, suggesting that these transcriptomic alterations may constitute a shared “extra-tumoral attribute” existing in both the early and advanced stages of NSCLCs. In line with this, a previous study using an A/J mouse-urethane model of human lung adenocarcinoma also identified a set of BAL cell-derived upregulated genes in the tumor-bearing lung segments at the early stage of tumor development; the expression levels of more than half of these genes turned out to be higher at a later time point as the tumors became more aggressive, with macrophage component of these mouse BAL cells appeared to be the major source of the gene signals (48). Similarly, a proteomic study of acellular BAL fluid by mass spectrometry from Carvalho et al. also showed an increase of cancer-specific protein biomarkers in the early stage of lung cancer and the expression remained increased in the advanced stage of the disease. However, as opposed to our transcriptomic study, neoplastic cells rather than immune cells appeared to constitute the major source of the differentially expressed proteins in the acellular BAL fluid (49). These results suggest; though adopting different approaches, the finding that a parallel evolvement of tumor body and peri-tumor immune reaction in alveolar milieu remains consistent across the studies, thus enables BAL an ideal measure for early diagnostic or prognostic biomarker discovery. However, caution should be taken as inconsistent source of biomarker signals can interfere biological interpretation across the studies, especially when different components of BAL samples were used for the investigations.

Of note, the characteristic signature, which contained 53 upregulated genes, showed significant positive correlations with the gene expression levels of multiple checkpoint proteins. This suggested that high expression of the signature may simultaneously be linked to the concept of adaptive immune resistance (50). These correlations were also confirmed in the published microarray data sets of early lung cancer, with the exception of the correlation for *CTLA-4*, an intriguing finding that likely indicates that the role CTLA-4 plays in peripheral tissues (as opposed to secondary lymphoid tissues) is less significant during early stage NSCLC. Whether or not this is a consequence of the lower tissue abundance of regulatory T cells in early stage cancer requires further investigation (51). As a whole, several genes of the signature reveal the relevance on tumor immunology and metabolic alteration of immune cells in tumor microenvironment. The overexpression of *CEACAM6*, which was very likely from the myeloid cells (e.g., macrophages, monocytes, and granulocytes), suggests that apart from its well-known involvement in the anoikis resistance of many epithelial carcinomas (52), further investigations of the role it plays in tumor-associated immune cells hold promise. In fact, the emerging role of CEACAM6 functioning as an immune checkpoint against cytotoxic T cells has been noted (53). In line with this, the genes *EPCAM, KRT7*, and *KRT19*, which are traditionally known to be responsible for some of the features of epithelial cells, have also been found to be characteristic of the circulating tumor-associated macrophages that frequently couple with cancer cells and function as the facilitator of cancer cell migration (54). *SPP1*, on the other hand, encodes a multifunctional secreted glycoprotein that is frequently expressed by tissue macrophages, and its engagement with integrin underlies the non-classical macrophage activation, angiogenesis, and migration of cancer cells (55). The upregulation of *MMP7* also underscores the significance of cell–matrix interactions in tumor development; however, its less consistent prognostic impact seen in earlier studies delays the elucidation of its role in the pathobiology of NSCLC (56, 57).

Several top genes of the signature, namely, *CPA3, TPSAB1*, and *TPSB2*, encode the carboxypeptidase A3 and tryptases that are highly abundant in tissue mast cells. Notably, mast cells have been found to accumulate around tumor blood vessels, and the release of these proteinases in collaboration with angiogenic factor VEGF has been found to be crucial for tissue remodeling, neovascularization, and immune suppression in tumor environments (58, 59). However, the prognostic effect of tumor-associated mast cells may be influenced by their relative localization, as the presence of mast cells within tumor islets as opposed to stroma was found to be a favorable feature in one recent study (60).

A set of seven genes of the signature encoding several structural units of immunoglobulin along with the overexpression of the B lymphocyte chemoattractant gene *CXCL13* suggests the significant role that humoral immunity plays during the development of NSCLCs. These findings are also backed by the prior work showing altered local immunoglobulin production in bronchial washing fluid collected from the lung segments in which lung cancer tumors are located as compared to the normal contralateral lung segments (61). Previous studies have reported the positive impact of *IGKC* on the survival of early stage NSCLC, in which stroma-infiltrating plasma cells were identified as the likely source of the *IGKC* (26). On the other hand, *IGJ, IGLJ3*, and *IGLC1* were found to be either characteristic or prognostic of a number of solid and hematologic malignancies (62–64). The coupling of these immunoglobulin genes with *YES1*, which encodes one of the Src-family kinases, potentially suggests that active signal transduction occurs as a result of engagement between the immunoglobulin and Fc receptors of macrophages or mast cells. In this regard, earlier studies showed that squamous carcinogenesis in murine models was found to involve the ligation of deposited circulating immune complex with the Fc receptors of tissue mast cells and macrophages (65, 66), and the deposition of circulating immune complex in tumor stroma was associated with tumor burden and poor prognosis (67).

It is notable that several top genes included in the signature gave insight into the metabolic perturbations of immune cells in tumor milieus, a finding that highlighted the emerging role of metabolic reprogramming of immune cells during cancer progression (68). *HS3ST2, CHST15*, and *GAL3ST4* are known to encode sulfotransferases for glycosaminoglycan and glycoprotein, with a recent study reporting the finding that sulfotransferase-mediated biosynthesis of cell-surface glycosaminoglycan may contribute to M2 macrophage polarization (69). Meanwhile, the overexpression of *SLC40A1*, which encodes an iron transporter ferroportin, has been found to be involved in the suppression of inducible nitric oxide synthase and impaired nitric oxide production in macrophages (70).

Altogether, the details of the signature indicate that multi-faceted functional pathways are exercised in the BAL cells of tumor-bearing lung segments and hint at multiple lines of study that could potentially be pursued. Clearly, a number of issues involving humoral and innate immunity are pivotal; this is underscored by our finding that not only the genes but the expression levels of the protein products IGKC, IGJ, and CPA3, when studied by immunohistochemistry, were also higher in the matrix of tumor adjacent normal lungs than in non-diseased lungs. Overall, one limitation of this study was the relatively small sample size; however, this concern was greatly alleviated by the utilization of the *in silico* measures using a wealth of published microarray datasets to verify the biological significance of our findings. On the other hand, the BAL cells from tumor-bearing lung segments were not directly compared with paired tumor adjacent normal lungs or tumor tissues; thus, the question of how representative their gene expression findings are for those of tumor-infiltrating immune cells remains unresolved, such that further investigations of the relationship between them are needed. This may also put forward the possibility of extrapolating the constitution of tumor-infiltrating immune cells from the BAL cells of NSCLC patients.

Above all, this study identified and validated the transcriptomic signature of BAL cells in the alveolar milieus of tumor-bearing lung segments. This signature, which has significant correlations with inhibitory checkpoints, not only is present in advanced NSCLCs but is also unique in the tumor adjacent non-neoplastic lung tissue at the early stage of lung cancers. IGKC and IGJ are biomarkers characterizing this transcriptomic profile, suggesting that the role of humoral immune responses in NSCLC development may warrant further study.

## Availability of Data

The transcriptomic data have been deposited in the Gene Expression Omnibus database, http://www.ncbi.nlm.nih.gov/geo (accession no. GSE103888 for gene expression data of BAL cells).

## Ethics Statement

The study was approved by the Ethics Committees of Chang Gung Memorial Hospital, Linkou, and all the participants gave the written informed consents in accordance with the Declaration of Helsinki.

## Author Contributions

We wish to acknowledge the individual contributions of each of the authors, which were as follows: conception and design: C-HK, C-YL, C-TY, and Y-KG; analysis and interpretation: C-HK, SP, T-HW, Y-WW, Y-LL, C-HC, K-YL, F-TC, T-YL, T-YW, and H-WK; drafting the manuscript for important intellectual content: C-HK and SP.

## Conflict of Interest Statement

The authors declare that the research was conducted in the absence of any commercial or financial relationships that could be construed as a potential conflict of interest.
