# Supplementary material for: Unique Immune Gene Expression Patterns in Bronchoalveolar Lavage and Tumor Adjacent Non-Neoplastic Lung Tissue in Non-Small Cell Lung Cancer
Source: Front Immunol. 2018 Feb 12;9:232. doi: 10.3389/fimmu.2018.00232 (PMC5816075; doi:10.3389/fimmu.2018.00232)
Supplement: Supplementary file 1 [file data_sheet_1.docx]

**Table S1: Primer pairs sequence for RT-qPCR**

| **No.** | **Name** | **Sequence** |
| --- | --- | --- |
| 1. | *CEACAM6* | Forward 5' CACAACCTGCCCCAGAATCGTAT 3'  Reverse 5' TTGGGCAGCTCCGGGTATACATG 3' |
| 2. | *IGKC* | Forward 5' CACAAAGTCTACGCCTGCGA 3'  Reverse 5' GGGGCACTTCTCCCTCTAAC 3' |
| 3. | *IGJ* | Forward 5' TCCTGGCGGTTTTTATTAAGGC 3'  Reverse 5' AGTAATCCGGGCACACTTACAT 3' |
| 4. | *SKAP2* | Forward 5' AGCAATCCACTAACAAGCAGTC 3'  Reverse 5' AGTGCTCTTATCCCCTGAGGT 3' |
| 5. | *SPP1* | Forward 5' CTCCATTGACTCGAACGACTC 3'  Reverse 5' CAGGTCTGCGAAACTTCTTAGAT 3' |
| 6. | *CXCL13* | Forward 5' GCTTGAGGTGTAGATGTGTCC 3'  Reverse 5' CCCACGGGGCAAGATTTGAA 3' |
| 7 | *SLC40A1* | Forward 5' TGGATGGGTTCTCACTTCCTG 3'  Reverse 5' GTCAATCCTTCGTATTGTGGCAT 3' |
| 8. | *CPA3* | Forward 5' GGGTTTGATTGCTACCACTCTT 3'  Reverse 5' GCCAAGTCCTTTATGATGTCTGC 3' |
| 9. | *YES1* | Forward 5' GGAAGCAAGATCAATCGCTACA 3'  Reverse 5' TCACCCCTTATCTCATCCCAAT 3' |

**Table S2: Transcriptomic signature of BAL cells of tumor-bearing lung segment**

| **SYMBOL** | **GENENAME** |
| --- | --- |
| *SPP1* | secreted phosphoprotein 1 |
| *CEACAM6* | carcinoembryonic antigen-related cell adhesion molecule 6 (non-specific cross reacting antigen) |
| *SLC40A1* | solute carrier family 40 (iron-regulated transporter), member 1 |
| *MAL2* | mal, T-cell differentiation protein 2 (gene/pseudogene) |
| *PEG3* | paternally expressed 3 |
| *ENPP6* | ectonucleotide pyrophosphatase/phosphodiesterase 6 |
| *IGHG3* | immunoglobulin heavy constant gamma 3 (G3m marker) |
| *CPA3* | carboxypeptidase A3 (mast cell) |
| *EPCAM* | epithelial cell adhesion molecule |
| *ZNF667-AS1* | ZNF667 antisense RNA 1 (head to head) |
| *SEPP1* | selenoprotein P, plasma, 1 |
| *SLITRK4* | SLIT and NTRK-like family, member 4 |
| *IGKC* | immunoglobulin kappa constant |
| *KRT19* | keratin 19 |
| *IGLJ3* | immunoglobulin lambda joining 3 |
| *IGLC1* | immunoglobulin lambda constant 1 (Mcg marker) |
| *IGLV@* | immunoglobulin lambda variable cluster |
| *CEACAM5* | carcinoembryonic antigen-related cell adhesion molecule 5 |
| *KRT7* | keratin 7 |
| *CBS* | cystathionine-beta-synthase |
| *HS3ST2* | heparan sulfate (glucosamine) 3-O-sulfotransferase 2 |
| *NQO2* | NAD(P)H dehydrogenase, quinone 2 |
| *SLC34A2* | solute carrier family 34 (type II sodium/phosphate contransporter), member 2 |
| *TFPI* | tissue factor pathway inhibitor (lipoprotein-associated coagulation inhibitor) |
| *CD24* | CD24 molecule |
| *RGL1* | ral guanine nucleotide dissociation stimulator-like 1 |
| *TPSAB1* | tryptase alpha/beta 1 |
| *TMEM37* | transmembrane protein 37 |
| *TPSB2* | tryptase beta 2 (gene/pseudogene) |
| *IGJ* | immunoglobulin J polypeptide, linker protein for immunoglobulin alpha and mu polypeptides |
| *SLC28A3* | solute carrier family 28 (concentrative nucleoside transporter), member 3 |
| *PLA2G7* | phospholipase A2, group VII (platelet-activating factor acetylhydrolase, plasma) |
| *CXCL13* | chemokine (C-X-C motif) ligand 13 |
| *TSPAN13* | tetraspanin 13 |
| *IGH* | immunoglobulin heavy locus |
| *GPRC5A* | G protein-coupled receptor, class C, group 5, member A |
| *RAB27B* | RAB27B, member RAS oncogene family |
| *OGFOD1* | 2-oxoglutarate and iron-dependent oxygenase domain containing 1 |
| *MYO10* | myosin X |
| *LOC642236* | FSHD region gene 1 pseudogene |
| *ZNF702P* | zinc finger protein 702, pseudogene |
| *ERBB3* | v-erb-b2 avian erythroblastic leukemia viral oncogene homolog 3 |
| *IGFBP4* | insulin-like growth factor binding protein 4 |
| *FAM20A* | family with sequence similarity 20, member A |
| *GPR116* | G protein-coupled receptor 116 |
| *SPRY2* | sprouty homolog 2 (Drosophila) |
| *BNIP3* | BCL2/adenovirus E1B 19kDa interacting protein 3 |
| *CHST15* | carbohydrate (N-acetylgalactosamine 4-sulfate 6-O) sulfotransferase 15 |
| *MERTK* | MER proto-oncogene, tyrosine kinase |
| *CTSW* | cathepsin W |
| *GAL3ST4* | galactose-3-O-sulfotransferase 4 |
| *ADORA3* | adenosine A3 receptor |
| *GJA1* | gap junction protein, alpha 1, 43kDa |
| *PNKD* | paroxysmal nonkinesigenic dyskinesia |
| *MUC1* | mucin 1, cell surface associated |
| *STEAP4* | STEAP family member 4 |
| *MMP7* | matrix metallopeptidase 7 (matrilysin, uterine) |
| *CCL13* | chemokine (C-C motif) ligand 13 |
| *SRGN* | serglycin |
| *ZNF415* | zinc finger protein 415 |
| *CCL2* | chemokine (C-C motif) ligand 2 |
| *SDS* | serine dehydratase |

**Figure S1**


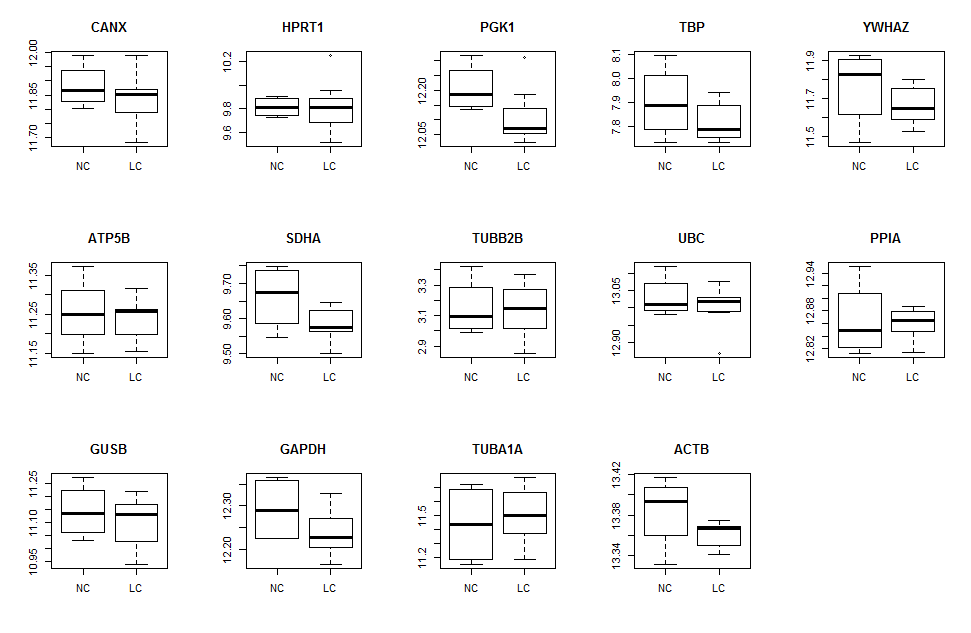


**#**

**Figure S2**


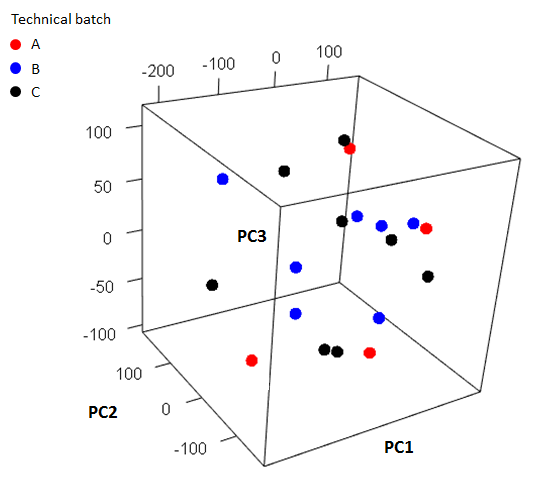


**Figure S3**


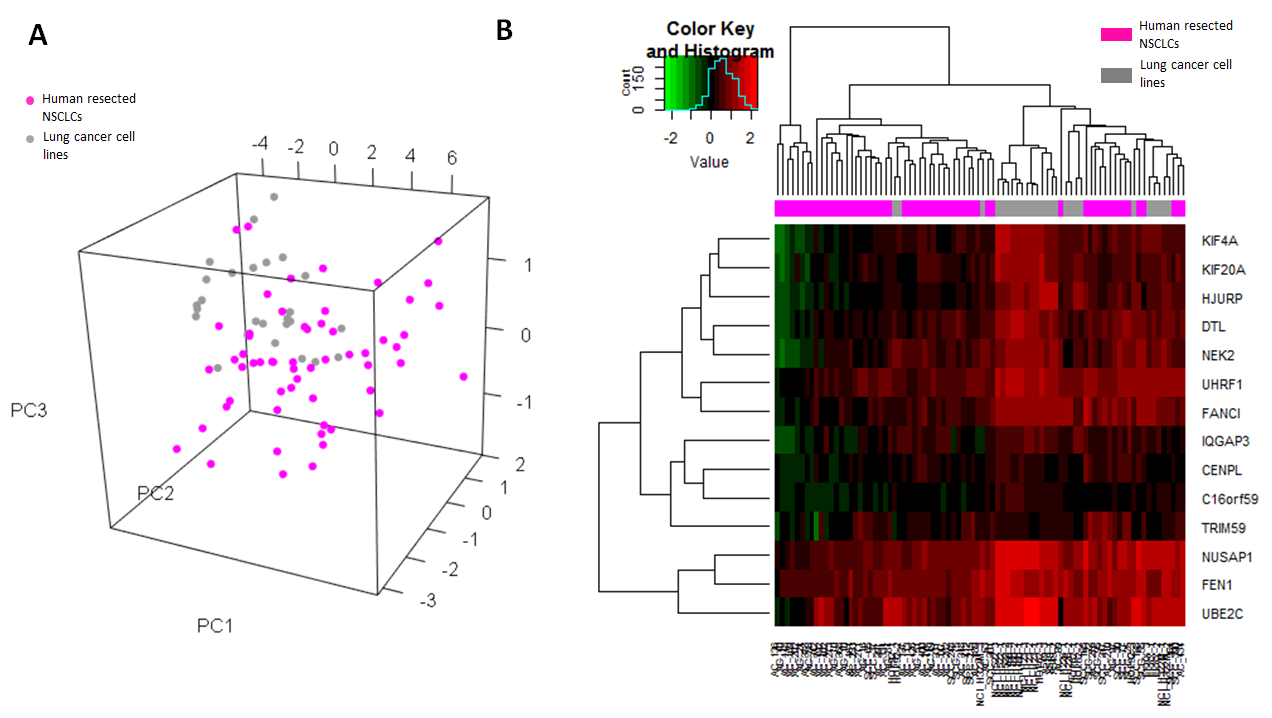


**Figure S4**

**Figure S5**


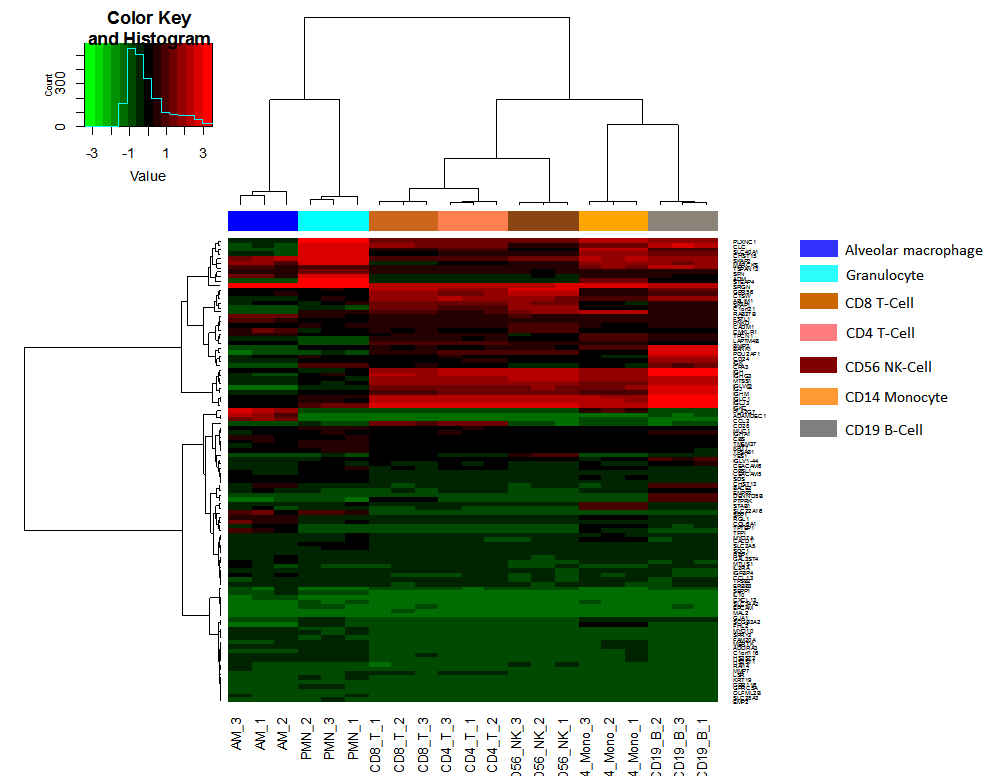


**Figure S6**

1. (B)


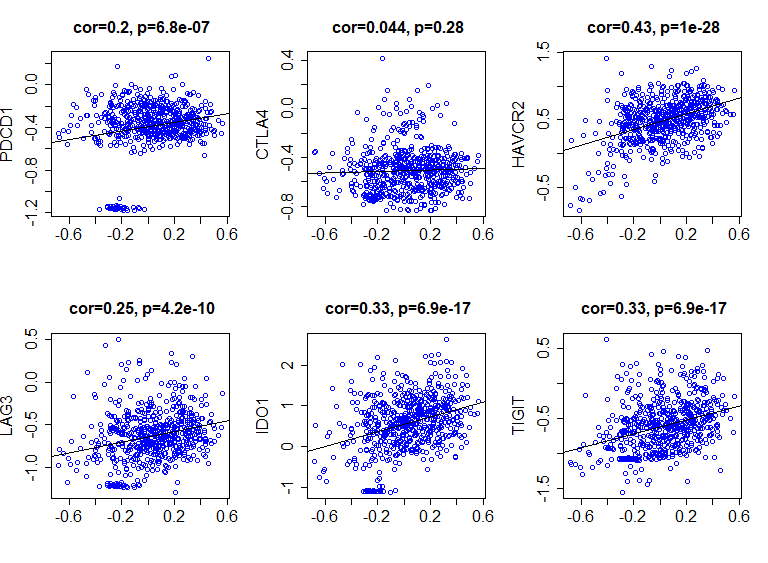

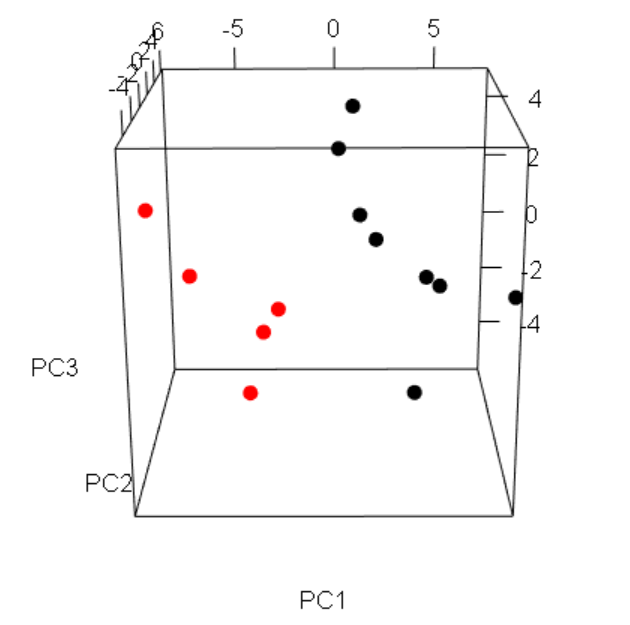


**Figure S7**


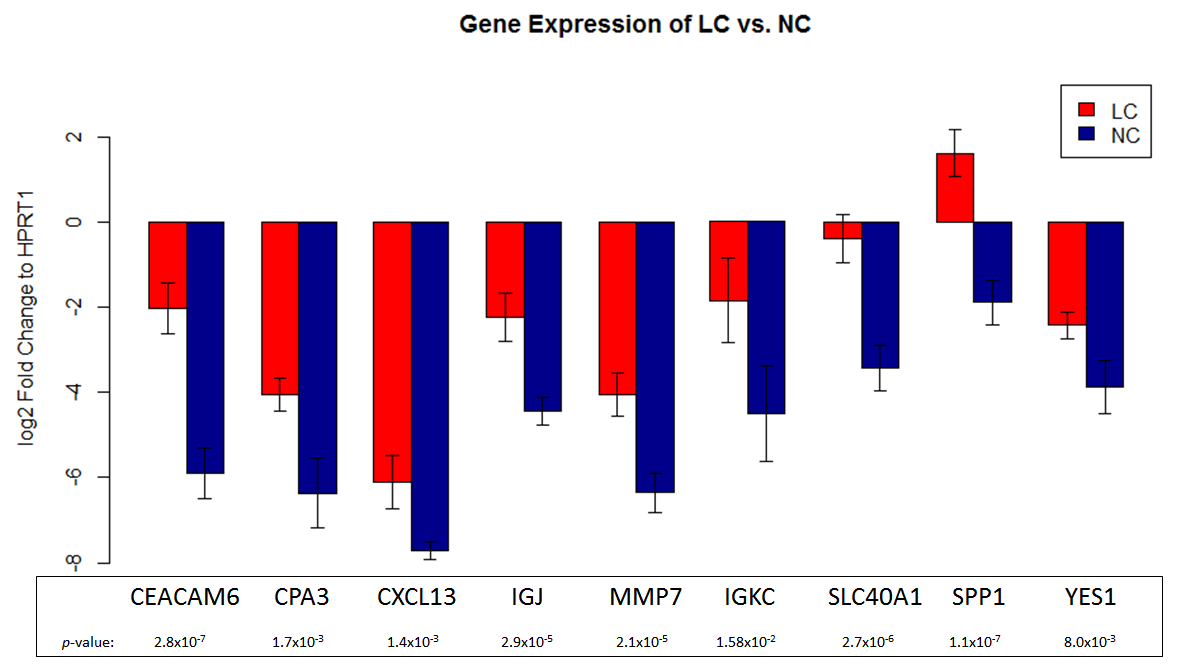


**Legends**

**Figure S1:** Box plots showing expression level from microarray for a set of 14 genes as candidate of endogenous reference. *HPRT1* was determined as endogenous reference for its constant expression between advance NSCLCs and healthy controls in the discovery group (#: *p*=0.984)

**Figure S2:** Visualization of the expression matrix by PCA from all microarray probe sets of the discovery group. Coloring scheme maps to the three technical batches

**Figure S3:** (A) TCGA 14-genes signature characteristic of malignant cells were extracted from the datasets GSE10245 (magenta) and GSE32474 (grey), which corresponded to the human resected NSCLCs and the 9 lung cancer cell lines of NCI-60 Human Tumor Cell Lines Panel, respectively. The standardized 14-genes expression matrix was visualized by PCA, where a dispersed pattern of distribution between the two datasets was noted. (B) Unbiased clustering of the same expression matrix failed to distinguish between resected tumors and cell lines.

**Figure S4:** Demonstration of the number of over-expressed genes found in normal human tissues by interrogation of the DEGs of BAL cellsusing dataset GSE1133.

**Figure S5:** Unbiased clustering using the standardized expression matrix of DEGs of BAL cellsextracted from GSE8823 (alveolar macrophage) and GSE72642 (non-alveolar macrophage) showed the cluster pattern mapped to the major subpopulations of immune cells.

**Figure S6:** (A) Correlation analysis of the signature of BAL cells (x-axis) with the gene expression of checkpoint protein PD-1/*PDCD1*, *CTLA4*, TIM-3/*HAVCR2*, *LAG3*, *IDO1* and *TIGIT* in the 8 published microarray datasets (n=607). Significantly correlations were noted except the one of *CTLA-4*. (B) PCA of the expression matrix of the signature from the 13 advanced NSCLC patients in the discovery group. Patients’ BAL cells showed a dichotomous gene expression pattern along the principle component (PC) 1 as delineated by the two subgroups colored by either red or black.

**Figure S7:** Log2 fold change of each of the 9 genes against *HPRT1* measured by RT-qPCR in the microarray discovery group between BAL cells of advanced NSCLC (red) and BAL cells of normal control (blue) subjects.
